# Switching the substrate specificity of lysoplasmalogen‐specific phospholipase D

**DOI:** 10.1002/2211-5463.13123

**Published:** 2021-03-19

**Authors:** Takayuki Oyama, Kazutaka Murayama, Daisuke Sugimori

**Affiliations:** ^1^ Materials Science Course Faculty of Symbiotic Systems Science and Technology Fukushima University Japan; ^2^ Division of Biomedical Measurements and Diagnostics Graduate School of Biomedical Engineering Tohoku University Sendai Japan

**Keywords:** lipoprotein‐associated phospholipase A_2_, LysoPAF‐specific PLD, lysoplasmalogen‐specific PLD, phospholipase D, substrate‐specificity switch, *Thermocrispum* sp.

## Abstract

Lysoplasmalogen‐specific phospholipase D (LyPls‐PLD) catalyzes reactions in a manner similar to those catalyzed by glycerophosphodiester phosphodiesterase (GDPD) and other well‐known PLDs. Although these enzymes hydrolyze the glycerophosphodiester bond, their substrate specificities are completely different. Previously, we reported that LyPls‐PLD from *Thermocrispum* sp. RD004668 shows only 53% activity with 1‐hexadecyl‐2‐hydroxy‐*sn*‐glycero‐3‐phosphocholine (LysoPAF) relative to the 100% activity it shows with choline lysoplasmalogen (LyPlsCho). Lipoprotein‐associated phospholipase A_2_ (Lp‐PLA_2_) activity can be used to evaluate for cardiovascular disease. Hence, development of a point‐of‐care testing kit requires a LysoPAF‐specific PLD (LysoPAF‐PLD) to measure Lp‐PLA_2_ activity. Rational site‐directed mutagenesis and kinetic analysis were applied to generate LysoPAF‐PLD from LyPls‐PLD and to clarify the mechanisms underlying the substrate‐recognition ability of LyPls‐PLD. Our results suggest that LyPls‐PLD variants A47, M71, N173, F211, and W282 are possibly involved in substrate recognition and that F211L may substantially alter substrate preference. Moreover, the specific activity ratio LysoPAF/LyPlsCho corresponding to F211L was up to 25‐fold higher than that corresponding to the wild‐type enzyme. Thus, we succeeded in switching from LyPlsCho‐ to LysoPAF‐PLD. These results suggest that the F211L variant may be utilized to measure Lp‐PLA_2_ activity. Kinetic analyses demonstrated that product release was the rate‐limiting step of the reaction, with flexibility of the *sn*‐1 ether‐linked vinyl/alkyl chain of the substrate being essential for substrate binding and product release. Our findings may lead to a better understanding of the differences between homologous enzymes (such as PLD, sphingomyelinase D, and GDPD of the phosphatidylinositol‐phosphodiesterase superfamily) in relation to substrate recognition.

**Enzyme:**

EC 3.1.4.2 (currently assigned).

## Graphical abstract

Lysoplasmalogen‐specific phospholipase D (LyPls‐PLD) intrinsically has a high affinity for choline lysoplasmalogen (LyPlsCho). By rational site‐directed mutagenesis (F211L), we converted LysPls‐PLD to a version (LysoPAF‐PLD) that has a high affinity for LysoPAF rather than the natural substrate LyPlsCho. This F211L variant of LyPls‐PLD exhibited a complete shift in substrate specificity with no decrease in catalytic efficiency or turnover number.

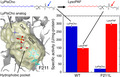

**Abbreviations**
Cho: choline
GDPD: glycerophosphodiester phosphodiesterase
Lp‐PLA_2_: lipoprotein‐associated phospholipase A_2_
LyPls: lysoplasmalogen(s)
LyPlsCho: 1‐*O*‐1'‐(Z)‐octadecenyl‐2‐hydroxy‐*sn*‐glycero‐3‐phosphocholine (choline lysoplasmalogen)
LyPls‐PLD: lysoplasmalogen‐specific phospholipase D
LysoPAF: 1‐hexadecyl‐2‐hydroxy‐*sn*‐glycero‐3‐phosphocholine
MSA: multiple sequence alignment analysis
PAF: platelet‐activating factor
PAF‐AH: platelet‐activating factor‐acetylhydrolase
PLA_2_: phospholipase A_2_
PLD: phospholipase D
SaPLD: phospholipase D from Streptomyces antibioticus
SMase D: sphingomyelinase D
WT: wild‐type enzyme

Lysoplasmalogen (LyPls)‐specific phospholipase D (LyPls‐PLD; currently assigned as EC 3.1.4.2) hydrolyzes choline LyPls (LyPlsCho) to choline (Cho) and 1‐(1‐alkenyl)‐*sn*‐glycero‐3‐phosphate (Fig. 1) [1]. We have previously reported that LyPls‐PLD (UniProt accession number A0A0U4VTN7) from *Thermocrispum* sp. RD004668 may be an ortholog of glycerophosphodiester phosphodiesterase (GDPD) [EC 3.1.4.46] [1], which is a member of the phosphoinositide‐specific phospholipase C (PLC)‐like phosphodiesterase superfamily (accession number cl14615), according to the NCBI Conserved Domain Database and of phosphatidylinositol (PI)‐phosphodiesterase superfamily (CATH superfamily 3.20.20.190). The PI‐phosphodiesterase superfamily also includes PI‐PLC [EC 3.1.4.11 and EC 4.6.1.13] and sphingomyelinase D (SMase D) [EC 3.1.4.41] [2]. On the other hand, well‐known PLD [EC 3.1.4.4] [3, 4, 5] is a member of endonuclease chain A superfamily (CATH superfamily 3.30.870.10) referring generally to as PLD superfamily, which contains CDP‐diacylglycerol‐serine *O*‐phosphatidyltransferases, cardiolipin synthases, a murine toxin and poxvirus envelope proteins, as well as specific and non‐specific endonucleases [6]. With the notable exception of substrate specificity, the catalytic reaction between GDPD and LyPls‐PLD is similar to that between GDPD and well‐known PLDs, the similarity being that these enzymes hydrolyze a phospholipid into its phosphatidate and a head group such as Cho. Currently, the differences between these enzymes in terms of function, substrate recognition, and biochemical roles remain unresolved.

**Fig. 1:**
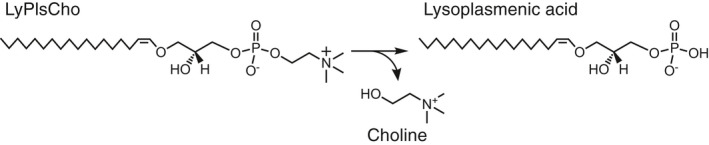
Hydrolysis of LyPlsCho by LyPls‐PLD.

A previous study of ours indicated that LyPls‐PLD shows 53% relative activity with 1‐hexadecyl‐2‐hydroxy‐*sn*‐glycero‐3‐phosphocholine (LysoPAF) than with LyPlsCho [7]. This increased activity level may be useful for conducting a lipoprotein‐associated phospholipase A_2_ (Lp‐PLA_2_) activity assay, as described below [8]. Platelet‐activating factor (PAF; 1‐*O*‐alkyl‐2‐acetyl‐*sn*‐glycero‐3‐phosphocholine) is a potent proinflammatory agent that displays a distinct spectrum of biological and pharmacological effects. In the cardiovascular system, PAF plays an important role in the initiation and progression of atherosclerosis and thus acts as an effective biomarker of coronary artery diseases [9, 10]. PAF is hydrolyzed to LysoPAF by PAF‐acetylhydrolase (PAF‐AH; EC 3.1.1.47), which belongs to the PLA_2_ family [11]. To date, three PAF‐AH isoforms have been identified: plasma PAF‐AH, intracellular type I PAF‐AH, and intracellular type II PAF‐AH. Plasma PAF‐AH is also known as Lp‐PLA_2_ due to its association with low‐density lipoproteins [9, 10, 12, 13]. Considering convenience and accuracy as well as high throughput, an Lp‐PLA_2_ activity assay, which does not involve radioisotopes or antibodies, may be preferable for measuring cardiovascular risk factors that help identify individuals at a higher risk for cardiovascular diseases [8]. Since 2010, several countries have advocated the use of Lp‐PLA_2_ assays for the purpose of evaluating cerebrovascular disease risks [14, 15, 16]. Currently, Lp‐PLA_2_ is measured by quantifying the protein concentration via an enzyme‐linked immunosorbent assay platform (Lp‐PLA_2_ mass assay) or directly measuring Lp‐PLA_2_ activity [17, 18, 19, 20, 21]. The most commonly used Lp‐PLA_2_‐activity assay in current clinical settings is the colorimetric assay, which uses 1‐myristoyl‐2‐(4‐nitrophenylsuccinyl) phosphatidylcholine (PAF analog) as the substrate (chemical Lp‐PLA_2_‐activity assay) [19, 21]. However, several issues are associated with this assay. For example, in addition to having low storage stability due to spontaneous degradation, PAF analogs may also be hydrolyzed by esterases in the serum. Therefore, the correlation between chemical assays and mass assays is not always high. Thus, more useful assays as well as enzymes, such as PLD, that show a preference for LysoPAF (LysoPAF‐PLD), which is released from PAF via the hydrolysis of Lp‐PLA_2_, may be required. To the best of our knowledge, such an enzyme has not yet been reported. Thus, converting LyPls‐PLD to LysoPAF‐PLD may enhance the efficacy of Lp‐PLA_2_‐activity assays. Besides, creation of LysoPAF‐PLD may provide important information regarding the mechanisms underlying substrate recognition and catalytic activity associated with LyPls‐PLD. In the present study, we generated a LysoPAF‐PLD from LyPls‐PLD via rational design and investigated the mechanisms underlying substrate recognition and catalytic activity of this LyPls‐PLD variant. Site‐directed mutagenesis of LyPls‐PLD allowed us to successfully generate LysoPAF‐PLD, without affecting the specificity of its activity. Additionally, the rate‐limiting steps associated with substrate recognition and catalysis of LyPls‐PLD were identified using steady‐state kinetic parameters. The results of steady‐state kinetics facilitated a better understanding of the differences between similar enzymes, such as GDPD, SMase D, and PLD, with regard to function, substrate recognition, and biochemical roles.

## Methods

### Materials

LyPlsCho (C18) and LysoPAF (C16) were purchased from Avanti Polar Lipids, Inc. (Alabaster, AL, USA). Bacto Tryptone was purchased from Becton Dickinson (Franklin Lakes, NJ, USA). The yeast extract, BSP‐B, and peroxidase (POD) were purchased from Oriental Yeast Co., Ltd. (Tokyo, Japan). Choline oxidase (COD) from *Arthrobacter globiformis* was provided by Asahi Kasei Pharma (Tokyo, Japan). 4‐Aminoantipyrine (4‐AA) was purchased from Nacalai Tesque Inc. (Kyoto, Japan). *N,N*‐Bis(4‐sulfobutyl)‐3‐methylaniline, disodium salt (TODB) was purchased from Dojindo Laboratories (Kumamoto, Japan). HisTrap HP columns (column volume: 5 mL) were purchased from GE Healthcare Japan (Tokyo, Japan). All other chemicals were of the highest purity and/or analytical grade.

Preparation of recombinant LyPls‐PLD [wild‐type (WT)] and assay of enzyme activity were conducted as reported previously [1]. A 50‐μL enzyme reaction mixture containing 0.965 U recombinant LyPls‐PLD (193 U·mL^−1^; 285 U·µg‐protein^−1^), 80 mm Tris/HCl buffer (pH 8.0), 0.4 mm substrate (LyPlsCho or LysoPAF), and 2 mm CaCl_2_ was incubated in a 1.5‐mL micro test tube at 50 °C for 1 min, following which the reaction was stopped by adding 5 μL of 0.1 m EDTA. The substrates were prepared as a micelle in ultrapure water. The concentration of released Cho was determined by using COD, POD, and modified Trinder's reagent with 4‐AA and TODB [1]. One unit (U) of enzyme activity was defined as the amount of enzyme that released 1 μmol Cho from the substrate per min.

### Rational site‐directed mutagenesis

The homology model of LyPls‐PLD was created as reported previously [1]. The docking model between LyPls‐PLD and LyPlsCho analog with a short chain (C5)‐alkenyl ether bond at *sn*‐1 was simulated using AutoDock [22] and drawn using Molfeat (v.5.2; FiatLux Corp., Tokyo, Japan). Based on the docking results, we predicted that A47, M71, N173, K177, F211, and W282 in LyPls‐PLD were likely involved in substrate recognition. We performed saturating mutagenesis at these sites via an inverse PCR (iPCR) with each primer set (Table S1) and using a recombinant plasmid vector, pET24, carrying the LyPls‐PLD gene (*lpls‐pld*) as a template (pET24/*lpls‐pld*). The primers were phosphorylated at 37 °C for 1 h; the phosphorylation reaction mixture (20 μL) contained 35 μm of each primer, Protruding End Kinase Buffer, 1 mm ATP, and 1 unit of T4 polynucleotide kinase (Toyobo Co., Ltd., Osaka, Japan). Following incubation for 5 min at 95 °C, the phosphorylated primers (10 μm) were used in the iPCR mixture (20 μL) containing KOD Fx Neo buffer (Toyobo Co., Ltd.), 6 pmol of each primer, 8 nmol of dNTPs, 0.08 U of KOD Fx Neo DNA polymerase, and 20 ng of pET24/*lpls‐pld* as a template. The thermal cycling parameters were 94 °C for 2 min, followed by 25 cycles of 98 °C for 10 s and 58 °C for 30 s, and finally 68 °C for 6.5 min. The purified iPCR products (10 μL) were treated with 20 U *Dpn* I (TaKaRa Bio Inc., Shiga, Japan) for 2 h at 37 °C, self‐ligated by adding an equivalent amount of Ligation high Ver. 2 (Toyobo), and incubated for 2 h at 16 °C. *Escherichia coli* DH5α competent cells (TaKaRa Bio) were transformed with the ligated DNA sample (1 μL) and selected on a Luria–Bertani (LB) agar plate containing 30 μg·mL^−1^ kanamycin. The pET24 vector DNA, having the desired mutation, was then transformed into a Zip Competent Cell BL21 (DE3; BioDynamics Laboratory Inc., Tokyo, Japan). The selected clones were cultured in 5 mL of LB broth containing 50 μg·mL^−1^ kanamycin (LBK) [1]. The cultured mutants were screened for enzyme activity towards the respective substrate; a 1% (v/v) inoculum of those showing activity was then transferred to 500‐mL flasks containing 100 mL of LBK, and cultivated at 37 °C while shaking (160 r.p.m.). After 6 h, 0.4 mm isopropyl‐β‐d‐thiogalactopyranoside was added and further incubated at 16 °C for 18 h (160 r.p.m.). The cultures were centrifuged (18 800 ***g***, 10 min) and washed thrice with 100 mL of 20 mm Tris/HCl buffer, pH 8.0 (buffer A). The resultant cell pellet was suspended in 30 mL of buffer A per 1 wet‐g of cells, disrupted on ice at 100 W output (20 kHz) for a total of 5 min using a TOMY UD‐201 sonicator (Tomy Seiko Co., Ltd., Tokyo, Japan), and subsequently centrifuged (21 800 ***g***, 10 min, 4 °C). The obtained supernatants were loaded onto a HisTrap HP column (column volume 5 mL), as reported previously [1]. The mutant enzymes were purified until electrophoretic homogeneity (Table 1 and Fig. S1), following which substrate specificity and kinetic parameters for each substrate were determined.

**Table 1:** Purification of LyPls‐PLD (WT) from recombinant *Escherichia coli* BL21(DE3).

Purification step	Activity^a^ (U·mL^−1^)	Sample (mL)	Protein (µg·mL^−1^)	Specific activity (U·µg‐protein^−1^)	Total activity (U)	% Recovery	Fold
CFE^b^	25.8	50	1.40	18.4	1290	100	1
HisTrap HP	193	1.0	0.680	285	193	15.0	18.8

^a^ WT activity was assayed at 37ºC for 1 min using a reaction mixture containing 80 mm Tris/HCl (pH 8.4), 2 mm CaCl_2_, and 0.4 mm LyPlsCho.

^b^ From 200 mL culture (3.95 wet‐g of cells).

### Enzyme kinetics

Steady‐state kinetics of mutant variants, which exhibited altered substrate specificity relative to that of WT, were investigated. For instance, the activity of A47G increased remarkably for LysoPAF, while remaining similar to the activity level for LyPlsCho, when compared to that of the WT; therefore, kinetic analysis was conducted only for LysoPAF. Besides, since W282 is highly conserved in SMase D and GDPD and plays important role in the catalytic reaction as mentioned in the discussion, we focused on this residue. Moreover, among W282 mutants, W282G was selected for kinetic analysis, since glycine has the smallest size and neutral residue.

**Fig. 2:**
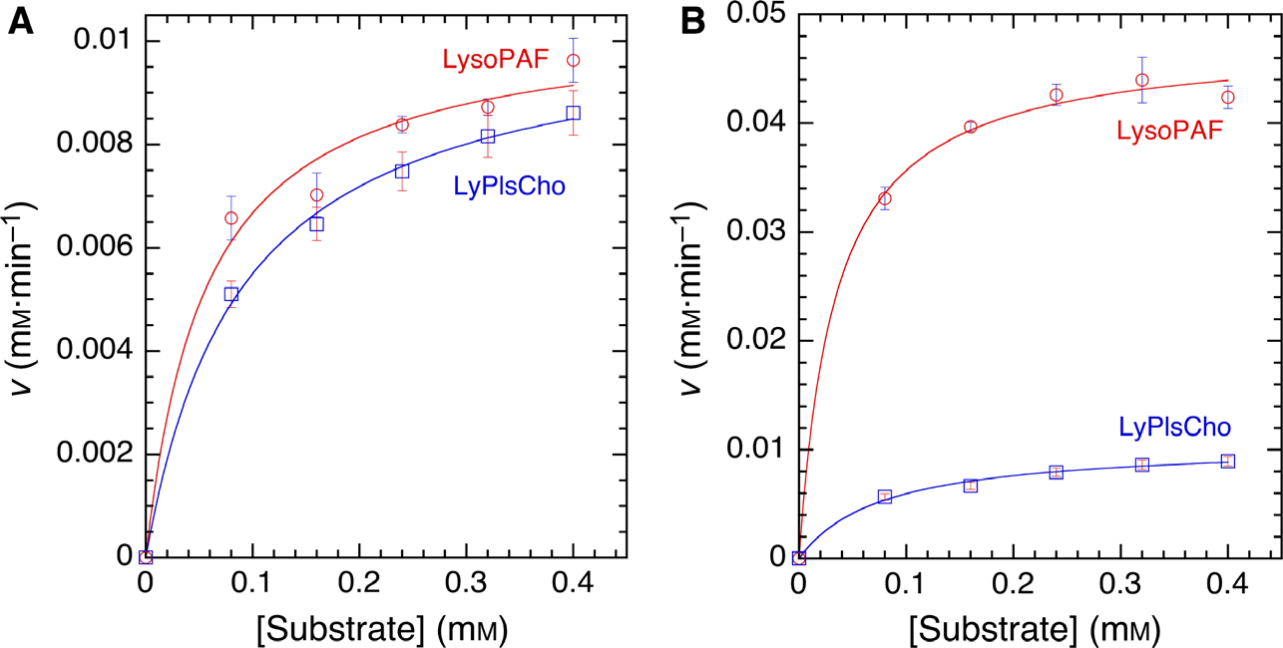
Representative Michaelis–Menten plots of WT (A) and F211L mutant (B). The initial velocity (*v*) of the enzyme reaction was assayed under standard assay condition. Data of experiments performed in triplicate are represented by the mean ± SD. Error bars represent SD (*n*, 3).

Initial velocity (*v*) of the enzymatic reaction was determined, for 1 min, at several substrate concentrations ([S]) under standard assay conditions, as reported previously [1]. Kinetic analyses were conducted at higher concentration than the critical micelle concentration of each substrate, which were 1–25 and 20 μm for LyPlsCho [23] and LysoPAF [24], respectively. The corresponding *v* vs [S] plot was evaluated using the Michaelis–Menten equation. Representative Michaelis–Menten plots are shown in Fig. 2. Due to enzymatic reactions in the substrate micelle system, the apparent kinetic constants *K*_m_^app^ and *k*_cat_^app^ were determined using nonlinear regression (KaleidaGraph; Synergy Software, Reading, PA, USA). Experiments were performed in triplicate, and data are represented by the mean ± standard deviation (SD).

## Results

### Substrate specificity of mutants

**Fig. 3:**
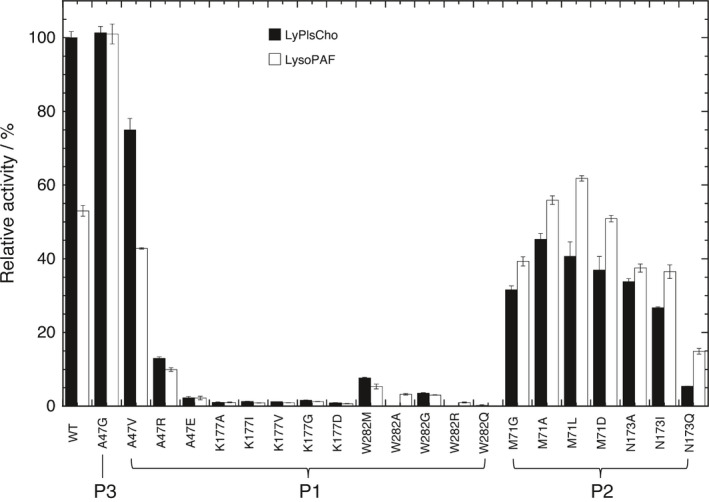
Substrate specificities of mutant enzymes. Relative activity was expressed by defining the specific activity (285 ± 5 U·µg‐protein^−1^) of WT towards LyPlsCho as 100%. Data of experiments performed in triplicate are represented by the mean ± SD. Error bars represent SD (*n*, 3).

**Fig. 4:**
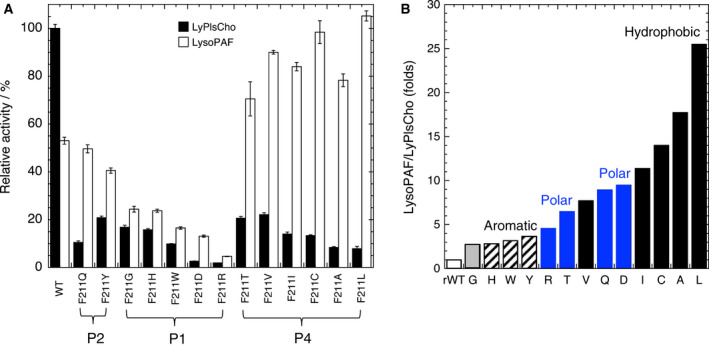
Substrate specificity (A) and selectivity (B) of F211 mutants. Relative activity was expressed by defining the specific activity (285 ± 5 U·µg‐protein^−1^) of WT towards LyPlsCho as 100%. Substrate selectivity, LysoPAF/LyPlsCho (units = fold change), represents the ratio of the specific activity toward each substrate. Data of experiments performed in triplicate are represented by the mean ± SD. Error bars represent SD (*n*, 3).

Several mutant enzymes (A47N/S, N173R, and K177), which produced variant enzymes that were in a nearly or completely inactive state, were successfully produced. Active mutant variants were divided into four patterns (patterns 1–4); (Figs 3 and 4A): Pattern‐1 included the A47(V/R/E), K177, W282, and F211(G/H/W/D/R) mutants whose activities with both LyPlsCho and LysoPAF, were uniformly and similarly reduced; Pattern‐2 included M71(G/A/L/D), N173(A/I/Q), and F211(Q/Y) mutants whose activities with LyPlsCho, particularly, were reduced, as well as M71 mutants, N173(A/I), and F211(Q/Y), which moderately hydrolyzed LysoPAF; Pattern‐3 contained A47G whose activity with LysoPAF was increased by approximately 1.5‐ to 2‐fold, while its activity with LyPlsCho was maintained at a constant level; and Pattern‐4 included F211(T/V/I/C/A/L) whose hydrolysis of LysoPAF was elevated, while activity with LyPlsCho was greatly reduced. Of all the variants, the F211 mutants showed interesting results related to substrate specificity, since all exhibited < 22% activity with LyPlsCho relative to the WT enzyme; (Fig. 4A). More specifically, the substrate specificity of F211L showing a preference for LysoPAF (i.e., LysoPAF‐PLD) was a complete reversal of that shown by WT. With regard to specificity, the F211L mutant showed twofold higher activity (300 ± 6 U·µg‐protein^−1^) with LysoPAF than the WT did, as well as 7.85 ± 0.92% and 105 ± 2% activity with LyPlsCho and LysoPAF, respectively. Substrate selectivity as determined by the ratio LysoPAF/LyPlsCho, which indicates specific activity with LysoPAF compared to that with LyPlsCho, increased after phenylalanine was replaced with other amino acids, in the order of smaller hydrophobic > polar or hydrophilic > aromatic residues (Fig. 4B). Finally, the increase in substrate selectivity of the F211L mutant was ~ 25‐fold that of WT, which was due to its very high specific activity.

### Steady‐state kinetics

Compared to WT, all M71 mutants showed increased substrate affinities (*K*_m_^app^) and decreased turnover numbers (*k*_cat_^app^) pertaining to LyPlsCho hydrolysis. However, the M71 mutations appeared to affect the turnover number more than substrate affinity, since *K*_m_^app^ and *k*_cat_^app^ were 1.4–1.9 and 1.5–5.6‐times lower, respectively (Table 2). Notably, M71D exerted the strongest negative effects on *K*_m_^app^ and *k*_cat_^app^. The apparent performance constant (*k*_cat_^app^ */K*_m_^app^) of M71L was somewhat increased while that of M71A was nearly similar to that of WT. Similarly, all tested N173 mutants showed substantially decreased substrate affinities and turnover numbers. As a result, *k*_cat_^app^ */K*_m_^app^ of all N173 mutants decreased. Likewise, substrate affinity and the turnover number of W282G declined substantially. All F211 variants, except F211Y, showed increased substrate affinity for LyPlsCho compared with WT while showing substantially decreased *k*_cat_^app^. Moreover, all M71, N173, F211, and W282 mutants showed substantially decreased turnover numbers for LyPlsCho, while the substrate affinities of these mutants were increased compared with that of WT. As a result, despite substrate affinity being increased compared with WT, almost all these mutants showed a reduced performance constant for LyPlsCho. On the other hand, many F211 variants displayed an increased performance constant for LysoPAF due to the main effect of *k*_cat_^app^ (Table 3).

**Table 2:** Steady‐state kinetic parameters for the hydrolysis of LyPlsCho by WT and mutant enzymes. The apparent kinetic parameters were determined under standard assay conditions (pH 8.0, 50 °C for 1 min). Data are represented by the means and SD of experiments performed in triplicate.

	*K*_m_^app^ (μm)		*k*_cat_^app^ (s^−1^)		*k*_cat_^app^ */K*_m_^app^ (mm ^−1^·s^−1^)	
WT	88.9 ± 8.76		122 ± 3.47		1371 ± 141	
M71G	64.8 ± 4.18	↓	57 ± 2.5	↓	880 ± 68.5	↓
M71A	59.4 ± 6.42	↓	83 ± 3.2	↓	1397 ± 160	≒
M71D	47.6 ± 13.1	↓	22 ± 1.3	↓	369 ± 76.5	↓
M71L	60.4 ± 12.0	↓	70 ± 3.8	↓	1501 ± 430	↑
N173A	200 ± 42.3	↑	42 ± 3.9	↓	212 ± 49	↓
N173I	169 ± 23.9	↑	51 ± 2.9	↓	303 ± 46.0	↓
N173Q	106 ± 15.3	↑	4.6 ± 0.21	↓	43 ± 6.60	↓
F211G	28.6 ± 8.42	↓	35 ± 3.3	↓	1221 ± 378	↓
F211A	42.4 ± 18.3	↓	40 ± 6.5	↓	943 ± 439	↓
F211V	55.0 ± 7.17	↓	45 ± 2.1	↓	818 ± 113	↓
F211C	21.2 ± 5.57	↓	21 ± 1.6	↓	972 ± 266	↓
F211T	36.6 ± 9.61	↓	31 ± 2.2	↓	844 ± 230	↓
F211D	37.1 ± 13.2	↓	8.8 ± 0.90	↓	238 ± 88	↓
F211L	76.7 ± 12.6	↓	21 ± 0.91	↓	271 ± 46.0	↓
F211Q	75.3 ± 1.32	↓	29 ± 0.14	↓	387 ± 7.00	↓
F211R	11.0 ± 1.81	↓	3.2 ± 0.12	↓	289 ± 49.0	↓
F211H	28.4 ± 5.49	↓	49 ± 2.6	↓	1733 ± 348	↑
F211Y	185 ± 22.2	↑	15 ± 0.78	↓	83 ± 11.0	↓
F211W	61.0 ± 5.11	↓	11 ± 0.19	↓	172 ± 15.0	↓
W282G	286 ± 51.7	↑	10.5 ± 0.95	↓	36.0 ± 7.25	↓

**Table 3:** Steady‐state kinetic parameters for the hydrolysis of LysoPAF by WT and mutant enzymes. The apparent kinetic parameters were determined under standard assay conditions (pH 8.0, 50 °C for 1 min). Data are represented by the means and SD of experiments performed in triplicate.

	*K*_m_^app^ (μm)		*k*_cat_^app^ (s^−1^)		*k*_cat_^app^ */K*_m_^app^ (mm ^−1^·s^−1^)	
WT	56.4 ± 16.9		74 ± 4.9		1303 ± 400	
A47G	4.44 ± 1.15	↓	98 ± 6.74	↑	2201 ± 589	↑
M71G	69.3 ± 10.2	↑	72 ± 2.9	≒	1041 ± 159	↓
M71A	78.7 ± 15.6	↑	55.4 ± 3.27	↓	703 ± 146	↓
M71D	93.9 ± 22.9	↑	96.4 ± 1.24	↑	1415 ± 70.8	↑
M71L	62.3 ± 10.5	↑	118 ± 1.94	↑	1823 ± 155	↑
N173A	110 ± 17.1	↑	106 ± 4.0	↑	965 ± 153	↓
N173I	101 ± 23.0	↑	44 ± 3.1	↓	437 ± 104	↓
N173Q	163 ± 46.1	↑	33 ± 3.7	↓	199 ± 61	↓
F211G	50.2 ± 16.5	↓	63 ± 7.5	↓	1262 ± 441	≒
F211A	45.1 ± 9.66	↓	149 ± 11	↑	3299 ± 744	↑
F211V	54.0 ± 3.73	↓	110 ± 1.6	↑	2046 ± 143	↑
F211C	52.2 ± 9.44	↓	207 ± 15	↑	3961 ± 769	↑
F211T	40.6 ± 5.49	↓	205 ± 8.9	↑	5064 ± 720	↑
F211D	70.8 ± 2.94	↑	33 ± 1.4	↓	472 ± 28	↓
F211L	26.8 ± 8.55	↓	187 ± 6.3	↑	6970 ± 2239	↑
F211Q	67.1 ± 1.77	↑	145 ± 14	↑	2164 ± 607	↑
F211R	44.7 ± 11.7	↓	14.0 ± 1.2	↓	311 ± 87	↓
F211H	38.7 ± 14.4	↓	112 ± 12.7	↑	2879 ± 1083	↑
F211Y	61.9 ± 8.20	↑	77 ± 2.0	≒	1248 ± 169	≒
F211W	56.3 ± 6.52	≒	4.4 ± 0.11	↓	79 ± 9.3	↓
W282G	71.7 ± 5.26	↑	4.4 ± 0.082	↓	60.9 ± 4.63	↓

Compared to those of WT, the substrate affinities of all M71 mutants, all N173 mutants, F211(D/Q/Y) mutants, and the W282G mutant toward LysoPAF were decreased, with particular reference to N173 mutants. In contrast, those of the A47G mutant and F211 mutants, except F211(D/Q/Y/W), were increased, with particular reference to A47G and F211L mutants (Table 3). Conversely, the turnover numbers of A47G, M71(D/L), N173A, and F211(A/V/C/T/L/Q/H) were increased compared with those of WT, particularly in F211(A/C/T/L/Q) mutants, whereas those of M71A, N173(I/Q), F211(G/D/R/W), and W282G decreased, particularly in F211W and W282G mutants. Compared to those of WT, the *k*_cat_^app^ */K*_m_^app^ values of A47G, M71(D/L), and F211(A/V/C/T/L/Q/H) were increased, with a parallel increase in the substrate affinity of F211(A/V/C/T/L/H). Specifically, F211(A/C/T/L/H) mutants showed substantially higher *k*_cat_^app^ */K*_m_^app^ values, relative to WT, implying a higher turnover number rather than a change in substrate affinity.

## Discussion

In this study, we used a point mutation to successfully switch the substrate specificity of PLD from LyPlsCho to LysoPAF without decreasing its activity. Notably, to the best of our knowledge, we are the first to create a LysoPAF‐PLD variant via rational design and mutagenesis. This LysoPAF‐PLD variant may be used as a diagnostic enzyme in Lp‐PLA_2_ assays [8]. However, further validation using human samples may be required. Our findings may help elucidate differences between the substrate‐recognition mechanisms of GDPD, SMase D, and PLD, as well as LyPls‐PLD.

**Fig. 5:**
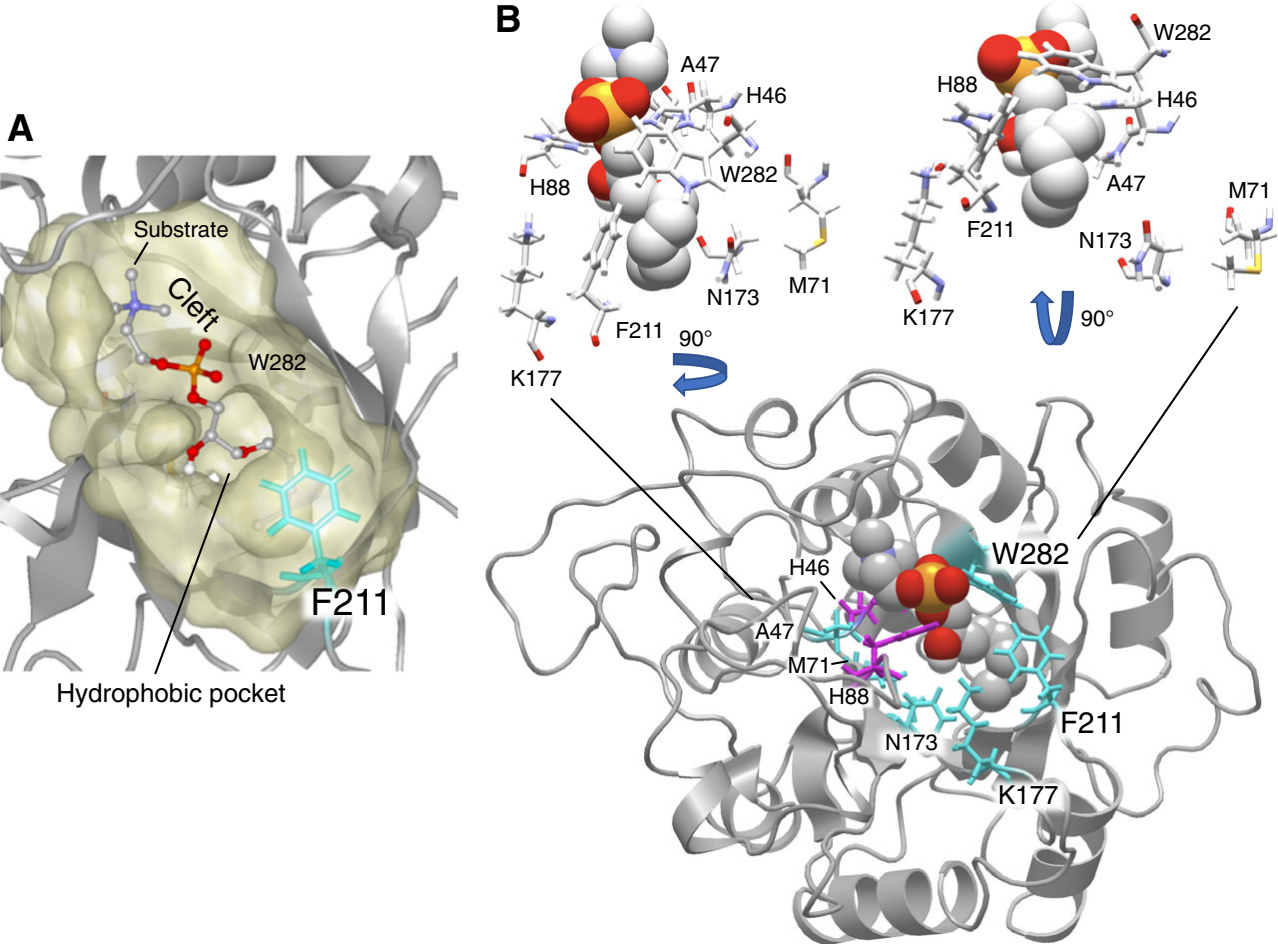
Models of the enzyme‐substrate complex. (A) Homology model of LyPls‐PLD docking with LyPlsCho analog (stick‐and‐ball) and surface representation of the predicted substrate‐binding cleft and pocket (yellow). (B) Amino acid residues composing the substrate‐binding cleft and pocket: Magenta indicates catalytic histidine; cyan represents amino acid residues presumed to be involved in the substrate binding and/or recognition.

We observed that point mutation altered substrate specificity according to four patterns. Replacement of rigid amino acids with smaller and more flexible variants (A47G, M71L, F211T/A/I/C/L) [25] increased hydrolytic activity with LysoPAF compared with that of WT. Steady‐state kinetics demonstrated that M71 mutation affected both *k*_cat_^app^ and *K*_m_^app^ (Tables 2 and 3), suggesting that its role in substrate binding as well as in product dissociation depended on its interactions with the *sn*‐1 carbon chain of the substrate. M71 was possibly localized proximally to the end of this chain (Fig. 5B), while N173, F211, and W282 were localized within the substrate‐binding pocket and cleft, resulting in an estimated ~ 3 Å distance between the closest nitrogen atom of N173 and the C12 methylene hydrogen of the *sn*‐1 carbon chain. The apparent performance constants, or *k*_cat_^app^ */K*_m_^app^ values, of all N173 variants for LysoPAF were higher than those for LyPlsCho. Specifically, N173A, which was different from other variants, reduced the *k*_cat_^app^ value for LyPlsCho hydrolysis by approximately three times, while increasing it for LysoPAF hydrolysis. Analyses of N173A/I suggested that hydropathy and the size of the substituted amino acid likely limited binding within the enzyme pocket. In fact, activity of the N173Q mutant with both substrates was substantially reduced due to its longer side chain. These results demonstrated that just one methylene group could remarkably affect both substrate binding and product release.

**Fig. 6:**
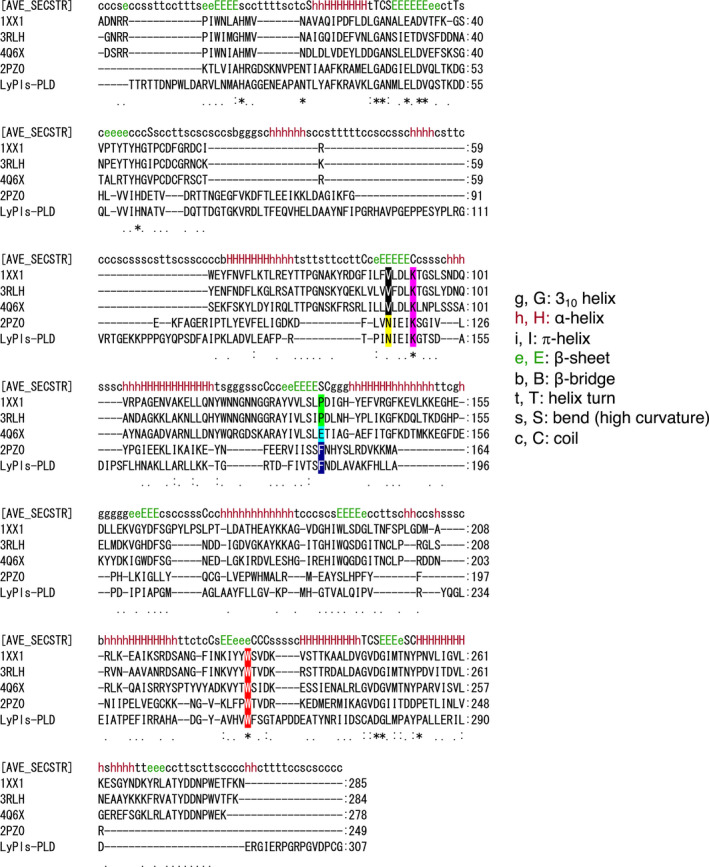
Alignment of LyPls‐PLD, SMase Ds, PLD, and GDPD using Multiple 3D Alignment software. Protein structures are indicated as follows: PDB‐ID, 1XX1; SMase D from *Loxosceles laeta*, 3RLH; *Loxosceles intermedia v*enom; 4Q6X, PLD from *Sicarius terrosus* venom, 2PZ0; GDPD from *Thermoanaerobacter tengcongensis*. Degrees of sequence similarities are shown as ‘average secondary structure’ (AVE_SECSTR). Completely and half, or second most conserved sites, are shown in upper and lower case letters, respectively. Each secondary structure is shown as in the DSSP program.

With reference to the K177 mutation, the nitrogen atom of the K177 ε amino group was located near the *sn*‐2 hydroxyl O atom and P atom of the substrate (Fig. 5B), suggesting that the K177 mutation strongly affected its activity. While validating this hypothesis, we found that the K177 mutants showed almost no activity (Fig. 3). Interestingly, multiple sequence alignment (MSA) demonstrated that both N173 and K177 were highly conserved in GDPDs, but not in PLDs (Fig. 6, highlighted in yellow and magenta) [1, 2].

**Fig. 7:**
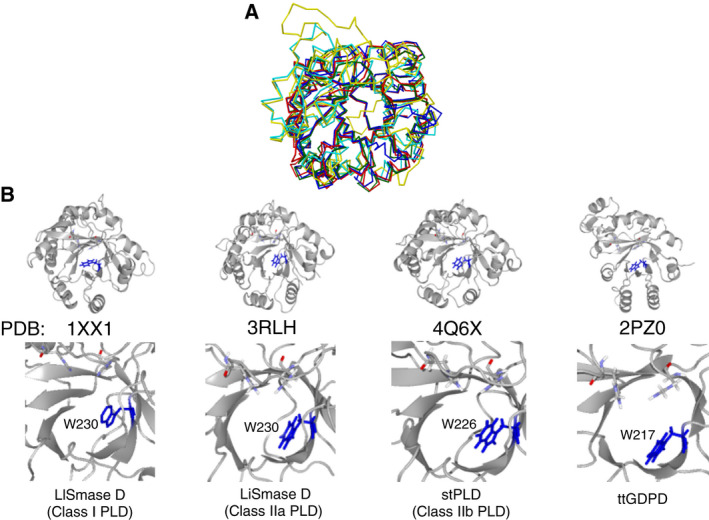
Crystal structural models of SMase Ds, PLD, and GDPD with highly conserved Trp. (A) Superimposition of predicted structural models of LyPls‐PLD (yellow) and crystal structural models of SMase D from *Loxosceles laeta* (LlSmase D, blue), SMase D from *Loxosceles intermedia* venom (LiSMase D, red), PLD from *Sicarius terrosus* venom (stPLD, green), GDPD from *Thermoanaerobacter tengcongensis* (ttGDPD, cyan). (B) The highly conserved Trp is depicted via blue stick models for each protein in (A), as well as their respective homologues.

**Fig. 8:**
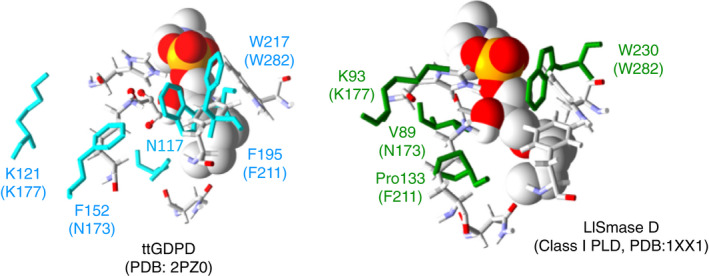
Superimposed LyPls‐PLD structural model and crystal structure of SMase D or GDPD. The structural model of LyPls‐PLD is shown as a stick model with atoms indicated in color. Ligand structure is depicted as a Van der Waals model. The amino acids in ttGDPD (GDPD from *Thermoanaerobacter tengcongensis*) and LlSMase D (SMase D from *Loxosceles laeta*) are indicated in cyan (left panel) and green (right panel), respectively.

The structural model indicated that the W282 residue formed a wall within the enzyme cleft (Fig. 5). The nitrogen atom of the W282 residue was estimated to be located within ~ 3 Å of the O atom of the phosphate group of the substrate analog. Multiple 3D alignment (http://strcomp.protein.osaka‐u.ac.jp/matras/index.html) demonstrated that the W282 residue of LyPls‐PLD corresponded to the W230 residue of SMase D from *Loxosceles laeta* (PDB: 1XX1) [2], the W230 residue of SMase D from *Loxosceles intermedia* venom (PDB: 3RLH) [2], the W226 residue of PLD from *Sicarius terrosus* venom (PDB: 4Q6X) [26], and the W217 residue of *Thermoanaerobacter tengcongensis* GDPD (ttGDPD, PDB: 2PZ0; Figs 7 and 8) [27]. In the present study, the W282 mutants, except W282G, showed almost no activity. Oliveira *et al*. reported that the above tryptophan was highly conserved in SMase D as well as in GDPDs, with a TIM‐barrel fold involved in substrate binding [28]. It has been suggested that this residue is involved in substrate stabilization via hydrogen bond formation between the N atom of tryptophan and the O atom of the substrate phosphate group [28]. However, tryptophan does not appear to be conserved in bacterial PLDs. We found that the W282G variant displayed decreased substrate‐binding affinity as well as a decreased turnover number, indicating that W282 may play the same role as the conserved tryptophan in SMase D and GDPDs. These results demonstrated that W282 was probably involved in substrate binding and product dissociation.

Almost all F211 variants reduced the performance constant, or specificity constant, for LyPlsCho even though the substrate affinities of these were increased compared with that of WT. However, the performance constants for LysoPAF were increased due to catalytic rates, suggesting that this enzyme reaction is dominated by *k*_cat_^app^ rather than *K*_m_^app^. Our conclusion was consistent with those of previous reports which stated that the second nucleophilic reaction, leading to final product release, was the rate‐limiting step [4, 5]. F211 was predicted to be located at the entrance of the hydrophobic pocket, into which the *sn*‐1 chain of the substrate enters (Fig. 5). The phenyl group of F211 was located ~ 4–5 Å away from several carbon atoms of the *sn*‐1 ether chain, as well as from the ether oxygen atom of the substrate analog. MSA and multiple 3D alignment demonstrated that location of F211 in LyPls‐PLD was reasonably unique, relative to its homologues GDPD, SMase D, and PLD, which exhibit F/I/P/S in GDPD, K/R in SMase D, and Q/L/R in PLD at this site, respectively [2, 4, 29, 30]. Although LyPlsCho contains a rigid vinyl ether‐linked alkyl chain at the *sn*‐1 position, LysoPAF possesses a flexible ether‐linked alkyl chain. The kinetic parameters of F211L allowed it to easily enter the substrate‐binding pocket of LysoPAF than that of LyPlsCho, likely due to the increased flexibility of this chain. However, such flexibility may also make it more difficult for the intermediate or product to dissociate from the catalytic pocket. This conclusion is supported by the fact that hydrolytic reaction with WT allowed the 100% cyclic product, 1‐hexadecyl‐2‐hydroxy‐*sn*‐glycero‐2,3‐cyclic‐phosphate, to be generated from LysoPAF, but not from LyPlsCho [7]. We also reported that the conformation of the *sn*‐1 chain of the substrate was likely involved in substrate binding and product release, which represented the rate‐limiting steps of the reaction, thereby suggesting that substrate recognition may be strongly influenced by the conformation of *sn*‐1. The specific volumes of leucine and phenylalanine were 166.7 and 189.9 Å^3^ [31], while the hydropathy indices were 3.8 and 2.8 [32], respectively. Moreover, isoleucine, leucine, and phenylalanine are all hydrophobic with large volumes [33], indicating that these characteristics may play a critical role in substrate recognition at this site. This further implied that replacing F211 with leucine may allow LysoPAF to easily enter the substrate‐binding pocket and release products, thereby increasing the turnover number relative to WT. This was supported by our findings pertaining to F211Y which showed that substrate binding and product dissociation were simply dependent on the presence or absence of only one OH group. Further structural analysis of LysoPAF‐PLD may be required in order to better understand the above results and elucidate the mechanisms responsible for LyPls‐PLD substrate recognition. We are currently engaged in solving the crystal structure of WT and LysoPAF‐PLD, that is, F211L variant, along with additional mutagenesis studies.

Considered together, we found that M71, N173, F211, and W282 played a role in substrate recognition by LyPls‐PLD. In particular, mutation of F211, which was predicted to be located at the entrance of the hydrophobic pocket into which *sn*‐1 chain of the substrate enters, resulted in a remarkable change in substrate specificity. Kinetic analyses demonstrated that product release was the rate‐limiting step of the reaction, with flexibility of the *sn*‐1 ether‐linked vinyl/alkyl chain of the substrate playing a significant role in both substrate binding and product release. Our findings may facilitate a better understanding of the differences between PLD and SMase D, as well as GDPD, with respect to substrate recognition. Moreover, effective evaluation of LysoPAF using the F211L variant may be useful for measuring Lp‐PLA_2_ activity during the assessment of cardiovascular and cerebrovascular disease risks.

## Conflicts of interest

The authors declare no conflict of interest.

## Author contributions

DS conceived and designed the project. TO acquired the data. DS and TO analyzed the data and wrote the paper. All authors discussed the results and read and approved the final manuscript.

## Supporting information


**Fig. S1.** SDS/PAGE analysis (12% gel) of cell‐free extracts (cfe) from BL21(DE3) recombinant cells and active fractions purified via HisTrap HP column (5 mL). (A) Lane 1 represents cfe (~ 21 µg protein) of the recombinant cells producing recombinant LyPls‐PLD (WT). (B) Active fractions (~ 0.2 to 0.5 µg protein) of WT purified from each respective cfe. (C) Lane 1′ represents cfe (~ 20 µg protein) of the recombinant cells producing the F211L mutant, and lanes 2′–6′ represent active fractions (~ 0.2 to 0.5 µg protein) of F211L mutant purified from its cfe. Lane M: molecular marker; Lane 1, 1′: cell‐free extracts of the BL21(DE3) recombinant cells producing WT and F211L mutant, respectively.

**Table S1.** List of iPCR primers used for generation of LyPls‐PLD variants.

## Data Availability

*Thermocrispum* sp. strain RD004668 was deposited as NITE BP‐01628 in the NITE Patent Microorganisms Depositary (NPMD; Chiba, Japan). The nucleotide sequences of 16*S* rRNA gene (accession number AB873024) and LyPls‐specific PLD gene (accession number AB874601) were deposited in the DDBJ database. The amino acid sequence of LyPls‐specific PLD (accession number A0A0U4VTN7) was deposited in the UniProt database.
