# Supplementary material for: Switching the substrate specificity of lysoplasmalogen‐specific phospholipase D
Source: FEBS Open Bio. 2021 Mar 19;11(4):1132–43. doi: 10.1002/2211-5463.13123 (PMC8016129; doi:10.1002/2211-5463.13123)
Supplement: Supplementary file 1 — Fig. S1. SDS/PAGE analysis (12% gel) of cell‐free extracts (cfe) from BL21(DE3) recombinant cells and active fractions purified via HisTrap HP column (5 mL). (A) Lane 1 represents cfe (~ 21 µg protein) of the recombinant cells producing recombinant LyPls‐PLD (WT). (B) Active fractions (~ 0.2 to 0.5 µg protein) of WT purified from each respective cfe. (C) Lane 1′ represents cfe (~ 20 µg protein) of the recombinant cells producing the F211L mutant, and lanes 2′–6′ represent active fractions (~ 0.2 to 0.5 µg protein) of F211L mutant purified from its cfe. Lane M: molecular marker; Lane 1, 1′: cell‐free extracts of the BL21(DE3) recombinant cells producing WT and F211L mutant, respectively. [file FEB4-11-1132-s001.pdf]

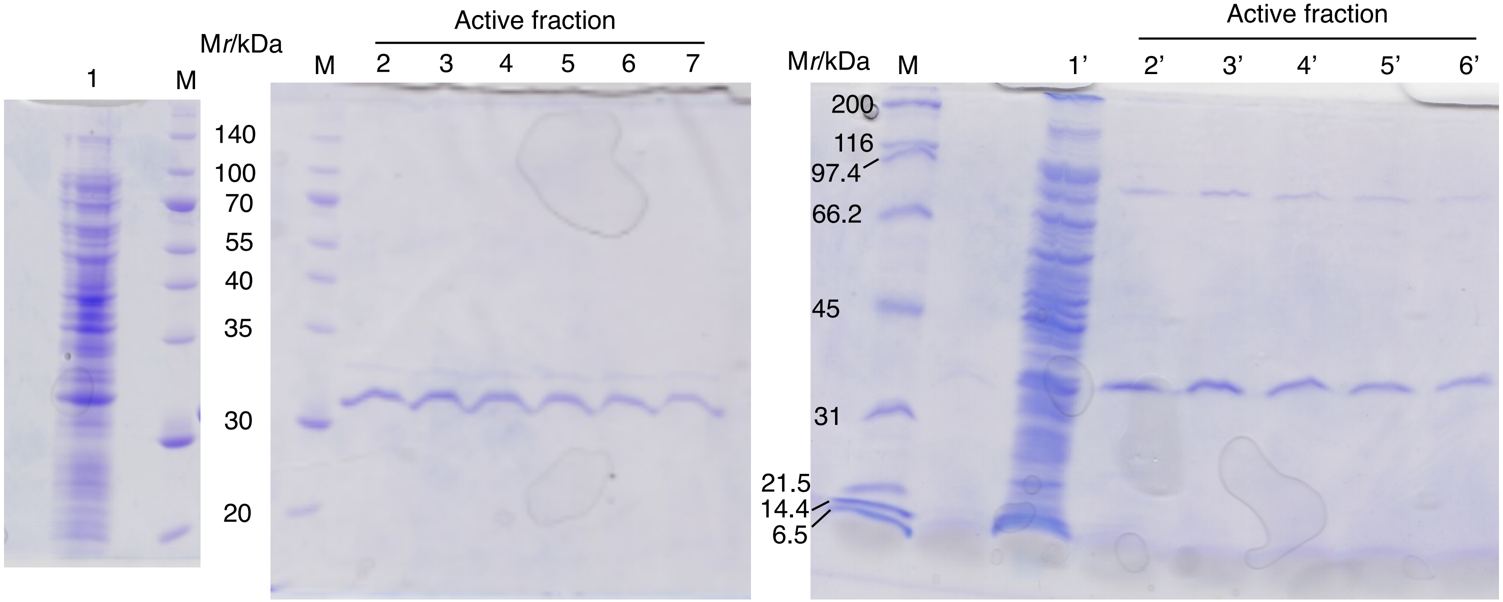

Fig. S1. SDS-PAGE analysis (12% gel) of cell-free extracts (cfe) from BL21(DE3) recombinant cells and active fractions purified via HisTrap HP column (5 mL)**.**

a) lane 1 represents cfe (~21 μg protein) of the recombinant cells producing recombinant LyPls-PLD (WT). b) Active fractions (~0.2 to 0.5 μg protein) of WT purified from each respective cfe. c) Lane 1' represents cfe (~20 μg protein) of the recombinant cells producing the F211L mutant, and lanes 2'-6' represent active fractions (~0.2 to 0.5 μg protein) of F211L mutant purified from its cfe.

Lane M: molecular marker

Lane 1, 1': cell-free extracts of the BL21(DE3) recombinant cells producing WT and F211L mutant, respectively.
