# Supplementary material for: Switching the substrate specificity of lysoplasmalogen‐specific phospholipase D
Source: FEBS Open Bio. 2021 Mar 19;11(4):1132–43. doi: 10.1002/2211-5463.13123 (PMC8016129; doi:10.1002/2211-5463.13123)
Supplement: Supplementary file 2 — Table S1. List of iPCR primers used for generation of LyPls‐PLD variants. [file FEB4-11-1132-s002.docx]

**Table S1. List of iPCR primers used for generation of LyPls-PLD variants.**

Underline represents the substituted codon.

| **Primer Name** | **Forward primers** | **Reverse primers** |
| --- | --- | --- |
| A47G | 5’-ggcggcggagaaaatgaagcg-3’ | 5’-gtgggccatgttgagcac-3’ |
| A47V | 5’-gtcggcggagaaaatgaagcg-3’ |  |
| A47S | 5’-tccggcggagaaaatgaagcg-3’ |  |
| A47E | 5’-gagggcggagaaaatgaagcg-3’ |  |
| A47R | 5’-cgcggcggagaaaatgaagcg-3’ |  |
| A47N | 5’-aacggcggagaaaatgaagcg-3’ |  |
| M71A | 5’-gcgctcgagctggatgtccaatcc-3’ | 5’-gttcgcaccgagctttaccgcgcg-3’ |
| M71G | 5’-gcgctcgagctggatgtccaatcc-3’ |  |
| M71D | 5’-gacctcgagctggatgtccaatcc-3’ |  |
| M71L | 5’-ctgctcgagctggatgtccaatcc-3’ |  |
| N173A | 5’-gcgatcgaaatcaaaggcaccagcgacgcg-3’ | 5’-gatcggcgttcgcgggaaggc-3’ |
| N173I | 5’-attatcgaaatcaaaggcaccagcgacgcg-3’ |  |
| N173Q | 5’-cagatcgaaatcaaaggcaccagcgac-3’ |  |
| N173R | 5’-cgtatcgaaatcaaaggcaccagcgac-3’ |  |
| K177A | 5’-gcaggcaccagcgacgcg-3’ | 5’-gatttcgatgttgatcggcgttcg-3’ |
| K177G | 5’-ggtggcaccagcgacgcg-3’ |  |
| K177D | 5’-gacggcaccagcgacgcg-3’ |  |
| K177I | 5’-attggcaccagcgacgcg-3’ |  |
| K177T | 5’-accggcaccagcgacgcg-3’ |  |
| K177V | 5’-gtgggcaccagcgacgcg-3’ |  |
| F211G | 5’-ggcaacgacctcgcggtg-3’ | 5’-cgacgtcacgatgaagtc-3’ |
| F211A | 5’-gcgaacgacctcgcggtggccaag-3’ |  |
| F211V | 5’-gtgaacgacctcgcggtg-3’ |  |
| F211C | 5’-tgcaacgacctcgcggtg-3’ |  |
| F211T | 5’-accaacgacctcgcggtg-3’ |  |
| F211D | 5’-gacaacgacctcgcggtg-3’ |  |
| F211L | 5’-ctgaacgacctcgcggtggccaag-3’ |  |
| F211I | 5’-attaacgacctcgcggtggccaag-3’ |  |
| F211Q | 5’-cagaacgacctcgcggtg-3’ |  |
| F211R | 5’-gcgaacgacctcgcggtg-3’ |  |
| F211H | 5’-actaacgacctcgcggtg-3’ |  |
| F211Y | 5’-tataacgacctcgcggtggccaag-3’ |  |
| F211W | 5’-tggaacgacctcgcggtggccaag-3’ |  |
| W282G | 5’-ggcttcagcggaacggcgccggac -3’ | 5’-cacgtgcaccgcgtagccgtcggc-3’ |
| W282A | 5’-gcgttcagcggaacggcgccggac -3’ |  |
| W282M | 5’-atgttcagcggaacggcgccggac -3’ |  |
| W282Q | 5’-cagttcagcggaacggcgccggac -3’ |  |
| W282R | 5’-cgcttcagcggaacggcgccggac -3’ |  |
